# The role of the gut microbiome in the progression of Parkinson’s disease: a systematic review of patient cohorts

**DOI:** 10.1007/s00415-025-13545-8

**Published:** 2025-12-06

**Authors:** Melissa Peters, Tobias Hegelmaier, Florian Wegner, Matthias Höllerhage, Lan Ye, Clara Niesmann, Ishana V. Schneidereit, Aiden Haghikia, Martin Klietz

**Affiliations:** Department of Neurology, Hannover Medical School, Carl-Neuberg-Straße 1, 30625 Hannover, Germany

**Keywords:** Parkinson’s disease, Gut microbiome, Dysbiosis, Prodromal Parkinson’s disease

## Abstract

**Introduction:**

Parkinson’s disease (PD) is the second most common neurodegenerative disease. The etiology of PD is not yet fully understood. In recent years, the role of the gut microbiome in the progression of the disease came to attention. A deeper understanding of the relationship between the gut microbiome and the development and progression of PD may innovate therapeutic approaches.

**Aim:**

The aim of the present literature analysis is to systematically evaluate alterations in gut microbiome in PD and its correlation with clinical symptoms.

**Materials and methods:**

A search for publications via PubMed using the search terms “Parkinson’s disease” AND “gut microbiome” AND “human” was performed. The main inclusion criteria were a subject number ≥ 30 per group, patients with clinically confirmed PD, an analysis of the gut microbiome in a case–control, cross-sectional or longitudinal study design.

**Results:**

The evaluation of the results showed that gut microbiome of PD patients is altered both in early stages of the disease and throughout its progression compared to healthy controls. These alterations correlate with clinical symptoms. In general, the diversity of micro-organisms in the gut is reduced in PD patients, and the composition of the gut microbiome differs significantly from healthy persons. Particularly a reduction in short-chain fatty acid (SCFA) producing genera such as *Faecalibacterium* and *Roseburia* and the increase in pro-inflammatory taxa such as *Collinsella* and *Akkermansia* is described.

**Conclusions and discussion:**

According to current evidence, the relationship between alterations in the gut microbiome and the pathogenesis of PD is not yet fully understood. Recent findings suggests that intestinal dysbiosis may contribute to the progression of PD.

**Supplementary Information:**

The online version contains supplementary material available at 10.1007/s00415-025-13545-8.

## Introduction

Parkinson’s disease (PD) is the second most common neurodegenerative disorder after Alzheimer’s disease, affecting approximately 400,000 people in Germany [1]. Globally, the number of PD patients increased from 5.5 million in 1990 to around 6.1 million in 2016, mainly due to an aging population [2].

PD is a chronic, progressive disorder characterized by the motor symptoms bradykinesia and at least one other symptom among tremor at rest or rigidity and in later stages postural instability, caused by the degeneration of dopaminergic neurons in the substantia nigra and resulting dopamine deficiency. The exact cause remains unknown [3]. Alpha-synuclein aggregation in Lewy bodies is one contributor to the pathogenesis [4]. Two subtypes of PD are described: brain-first (initial alpha-synuclein deposition in the brain) and body-first (initial deposition in the peripheral nervous system, possibly originating in the gut and influenced by the microbiome and immune system and spreading via the vagus nerve or through the blood) [5]. Non-motor symptoms such as REM sleep behavior disorder (RBD), gastrointestinal issues, and depression can precede motor symptoms by years [6]. While motor symptoms can be managed with dopaminergic therapy, the disease remains incurable [4].

The gut microbiome consists of trillions of micro-organisms, mainly bacteria, but also viruses and fungi, inhabiting the gastrointestinal tract. It is distinct from the term microbiota, which refers to the microbes themselves. The gut microbiome harbors over 3 million genes, compared to around 23,000 in the human genome, and is often referred to as a “virtual organ”. Microbiome composition is influenced by genetics, environment, and lifestyle factors, such as diet, medication, stress, and smoking [7]. Its functions include fermenting indigestible substrates, producing short-chain fatty acids (SCFAs), and synthesizing hormones and metabolites. It also plays a crucial role in immune regulation by maintaining gut barrier integrity and influencing immune cell development [8].

Disruption of this balance, known as dysbiosis, can result from infections, antibiotics, or other stressors and may contribute to chronic diseases, including autoimmune and neurodegenerative disorders [9].

Understanding the role of the gut microbiome in PD could facilitate the development of novel diagnostic and therapeutic approaches. Several hypotheses propose a potential link between the gut and the pathogenesis of PD. Braak’s hypothesis, in particular, suggests that PD may originate in the gastrointestinal tract, with pathological alpha-synuclein propagating from the gut to the brain via the vagus nerve [10]. The aim of this systematic review is to examine alterations in the microbiome during the early and late stages of PD and to assess their correlation with clinical manifestations.

## Materials and methods

**Fig. 1:**
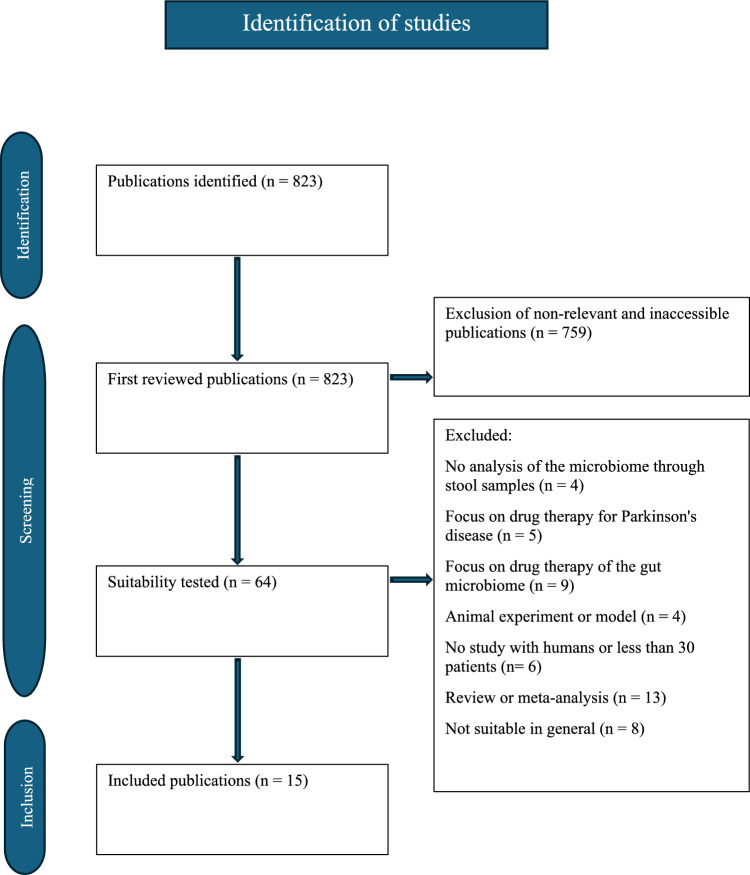
Flowchart of the process of literature research

Data collection for the evaluation of relevant literature was conducted via the meta-database PubMed. The main research question was defined as: “How does the gut microbiome change in PD, including also prodromal stages, and how does it correlate with clinical symptoms and progression of the patients?” Only studies with a minimum of 30 patients per group were included. Eligible study designs comprised case–control, cross-sectional or longitudinal studies. Patients were required to have a confirmed diagnosis of PD or to be in a prodromal stage of the disease and not receiving any microbiome-targeted therapy. Analysis of the gut microbiome had to be based on stool sample collections. Conversely, reviews and meta-analyses were excluded, as well as studies without human subjects, such as animal experiments or model-based studies. Only articles published up to February 27th, 2025, were considered. The search for relevant studies was conducted using the terms “Parkinson’s disease”, “gut microbiome”, and “human”. The operator “AND” was used to combine the search terms. Since the terms were applied in a single combined order, no duplicates were present in the search results. Using the previously mentioned terms 823 publications were found. Initially, unavailable studies were excluded by applying an availability filter (*n* = 759). Subsequently, studies were manually screened by title and abstract regarding the research questions and the predefined inclusion and exclusion criteria. Studies deemed suitable during this process were then assessed in full-text form (*n* = 64). Only those studies meeting all criteria were ultimately included in this systematic review (*n* = 15). This process is summarized in Fig. 1. All included studies were systematically assessed by the QUADAS-2 score for the risk of bias (Supplementary Table 1) [11]. The systematic review was conducted in accordance with the PRISMA guidelines for systematic reviews [12].

## Results

A total of fifteen studies were considered that met the above criteria and were deemed suitable for analysis. Each of the included studies addressed both PD and the gut microbiome.

### Alterations of the Gut microbiome in the early stages of Parkinson’s disease

The following section presents published data on alterations of the gut microbiome in the early stages of PD. REM sleep behavior disorder represents a key symptom of prodromal PD and is discussed separately. In these studies, the authors aim to present results of alterations in the gut microbiome in early stages of PD and how these alterations change in the progression of the disease. The most important results are summarized in Table 1.

**Table 1:** Studies on changes in the gut microbiome in the early stages of Parkinson’s disease

Study	Region	Participants	Methods	Results and discussion
Palacios et al. [13]	USA	*n* = 420 (early PD (*n* = 75), prodromal PD (*n* = 101), controls with obstipation (*n* = 113), healthy controls (*n* = 131))	Deep shotgun sequencing	Significantly reduced abundance of several bacterial species (*Roseburia faecis*, *Eubacterium rectale*, *E. ramulus*, *Oscillibacter_sp_57_20*, *Bacteroides xylanisolvens* and *E. siraeum*) Increased abundance of *Bifidobacterium dentium*, *B. longum*, *E. tayi*, *C. leptum* and *R. lactatiformans* Significantly impaired microbial metabolic pathways related to carbohydrate metabolism Shift toward a pro-inflammatory milieu
Huang et al. [14]	Hong Kong	*n* = 441 (RBD-FDR (*n* = 127), RBD (*n* = 170), early PD (*n* = 36), healthy controls (*n* = 108))	16S rRNA sequencing	Overall microbiota composition in RBD patients closely resembles that of early-stage PD (characterized by a deficiency of butyrate-producing bacteria and an overabundance of *Collinsella*, *Desulfovibrio*, and *Oscillospiraceae* UCG-005) General increase in fatty acid fermentation toward lactate and ethanol, accompanied by decreased levels of deazapurine biosynthesis
Zhang et al. [16]	China	*n* = 189 (de novo PD with RBD (*n* = 30), de novo PD without RBD (*n* = 64), RBD (*n* = 35), healthy controls (*n* = 60))	16S rRNA sequencing	Beta diversity showed significant differences Patients with RBD, PD with RBD and PD without RBD exhibited as *Ruminococcus*-dominant, whereas healthy controls were characterized by *Bacteroides* dominance Seven genera were consistently altered in both RBD and PD with RBD, with four showing a significant increase (*Aerococcus*, *Eubacterium*, *Gordonibacter*, and *Stenotrophomonas*) and three showing a significant decrease (*Butyricicoccus*, *Faecalibacterium*, and *Haemophilus*)
Heintz-Buschart et al. [15]	Europe	*n* = 175 (PD (*n* = 76), RBD (*n* = 21), healthy controls (*n* = 78))	16S and 18S rRNA sequencing	Increased abundance of *Akkermansia* (enrichment of *Akkermansia muciniphila* correlates with prolonged intestinal transit time) Differential abundance of the bacterial family *Prevotellaceae*

This table describes the main findings of the studies on changes on the gut microbiome in the early stages of PD that include prodromal PD and REM sleep behavior disorder (RBD). The study by Huang et al. also includes a group with first degree relatives from patients with RBD (RBD-FDR)

*PD* Parkinson’s disease, *RBD REM* sleep behavior disorder, *RBD-FDR REM* sleep behavior disorder first degree relatives, *USA* United States of America

#### Prodromal Parkinson’s disease

The case–control study (*n* = 420) by Palacios et al. from the United States examined the gut microbiome of both PD patients and individuals showing signs of prodromal PD, compared with two control groups (patients with constipation and healthy individuals). Based on questionnaire responses addressing symptoms such as constipation, reduced sense of smell, and probable REM sleep behavior disorder participants were assigned to the prodromal PD (PPS) group. The researchers observed a significantly reduced abundance of several bacterial species in PD samples compared to healthy controls, including *Roseburia faecis*, *Eubacterium rectale*, *E. ramulus*, *Oscillibacter* sp. 57_20, *Bacteroides xylanisolvens,* and *E. siraeum*. Moreover, the reduced abundance of these species followed a gradient across most analyses, progressing from healthy controls to constipation controls, PPS, and finally PD. An increased abundance of *Bifidobacterium dentium* and *B. longum* was also observed in the PPS group. In addition, *E. tayi*, *C. leptum*, and *R. lactatiformans* were found in higher abundance in samples from recently diagnosed PD patients compared to healthy controls, with a stepwise increase observed from healthy controls to constipation controls, PPS, and early PD. Significantly impaired microbial metabolic pathways related to carbohydrate metabolism were also identified. When comparing the main microbiome alterations observed in PPS, the authors noted that species over- or under-represented in PD compared to healthy individuals showed similar associations in PPS. The overall abundance of previously defined anti-inflammatory species was lower in PD compared to both healthy and constipation controls and showed a gradual decline across study groups, with the highest levels in healthy controls and the lowest in PD patients. These findings suggest a shift toward a proinflammatory microbial profile in PD. In summary, the study illustrates how the gut microbiome in individuals with prodromal PD lies on a continuum between healthy controls and PD patients [13].

#### REM sleep behavior disorder

In the study (*n* = 441) by Huang et al. from Hong Kong, the researchers investigated the gut microbiome of patients in the early stage of PD, patients with RBD, their first-degree relatives (RBD-FDR), and a control group. They found that the overall microbial composition in RBD patients closely resembled that of early-stage PD, characterized by a depletion of butyrate-producing bacteria and an overabundance of *Collinsella*, *Desulfovibrio*, and *Oscillospiraceae* UCG-005. In RBD-FDRs, representing younger cohort, RBD/PD-like microbial alterations were also observed, particularly the increase in pro-inflammatory *Collinsella* and the reduction in butyrate-producing taxa. The predicted functional profile showed an overall increase in fatty acid fermentation to lactate and ethanol, along with reduced levels of deazapurine biosynthesis in RBD-FDRs, RBD patients, and early PD patients. In addition, the authors identified host factors such as stool frequency (also acting as a mediator), sex, age, and the use of medications (e.g., antidepressants, statins, and osmotic laxatives) as partial contributors to microbial shifts in RBD-FDRs, RBD, and early PD. In summary, this study suggests that gut dysbiosis may occur at a much earlier stage, prior to the onset of both RBD and PD, highlighting the potential role of the gut microbiome in the pathogenesis of PD [14].

In another study (*n* = 175) conducted in Europe, the nasal and gut microbiomes of patients with RBD, PD, and healthy controls were compared. In PD patients, a higher abundance of *Akkermansia* was observed compared to healthy controls. Enrichment of *Akkermansia muciniphila* correlated with longer intestinal transit time. The abundance of *Prevotellaceae* differed between PD and RBD patients. No major differences were detected in the nasal microbiome. Overall, this study identified several compositional differences in the gut microbiome between PD, RBD, and healthy individuals [15].

The aim of another study (*n* = 189) from China was to investigate whether consistent gut microbiome alterations exist between RBD and PD. To this end, de novo PD, RBD patients, and healthy controls were recruited. PD patients were further divided into those with and without RBD. Several specific microbial biomarkers were identified in RBD that may indicate progression toward PD. Alpha diversity, a measure for species richness within a sample, showed no significant differences, whereas beta diversity, a measure for differences in species composition between samples, revealed notable group-specific distinctions. The microbiota composition revealed that RBD, PD with RBD, and PD without RBD were predominantly *Ruminococcus*-dominated, whereas healthy controls were *Bacteroides*-dominated. Seven genera showed consistent changes in RBD and PD with RBD: four were significantly increased (*Aerococcus*, *Eubacterium*, *Gordonibacter*, and *Steno-trophomonas*) and three were significantly decreased (*Butyricicoccus*, *Faecalibacterium*, and *Haemophilus*). Among these, four genera (*Aerococcus*, *Eubacterium*, *Butyricicoccus*, and *Faecalibacterium*) remained distinguishable between PD with RBD and PD without RBD. Clinical correlation analysis showed that *Butyricicoccus* and *Faecalibacterium* were negatively correlated with RBD severity. Functional analysis revealed that RBD exhibited similarly elevated staurosporine biosynthesis as PD with RBD. The study suggests that RBD shares similar gut microbial alterations with PD. In summary, a reduced abundance of *Butyricicoccus* and *Faecalibacterium* may serve as potential microbial signature of phenoconversion from RBD to PD [16].

### Alterations of the gut microbiome in the manifest stage of Parkinson’s disease

The following section addresses gut microbiome alterations in advanced stages of PD. Table 2 provides an overview of the results. These studies focus on changes in the gut microbiome between PD patients and healthy controls and aim to evaluate how gut dysbiosis and inflammation may play a role in PD pathogenesis.

**Table 2:** Studies on changes in the gut microbiome in the manifest stages of Parkinson’s disease

Study	Region	Participants	Methods	Results and discussion
Zhang et al. [20]	USA	*n* = 170 (PD (*n* = 96), healthy controls (*n* = 74))	16S rRNA sequencing	Significantly reduced microbial diversity and a significantly altered microbiota composition Increased abundance of three bacterial phyla (*Proteobacteria*, *Verrucomicrobiota, and Actinobacteria*) and five bacterial genera (*Akkermansia*, *Enterococcus*, *Hungatella*, and two *Ruminococcaceae genera*)
Mehanna et al. [21]	Egypt	*n* = 65 (PD (*n* = 30), healthy controls (*n* = 35))	16S rRNA sequencing	Significant increase in *Bacteroides*, along with a significant decrease in *Firmicutes*, the *Firmicutes*/*Bacteroidetes* ratio, and *Bifidobacteria*
Wallen et al. [19]	USA	*n* = 724 (PD (*n* = 490), healthy controls (*n* = 234))	Deep shotgun sequencing	More than 30% of the tested microbiome parameters showed significant differences The metagenomic profile observed in PD suggests the presence of a microbiome with disease-promoting characteristics
Wallen et al. [18]	USA	Data set 1: * n* = 316 (PD (*n* = 199), healthy controls (*n* = 117)); Data set 2: * n* = 486 (PD (*n* = 312), healthy controls (*n* = 174))	16S rRNA sequencing	Description of the SNCA gene as a potential key player in the pathogenesis of PD
Wallen et al. [17]	USA	Data set 1: * n* = 348 (PD (*n* = 212), healthy controls (*n* = 136)); Data set 2: * n* = 507 (PD (*n* = 323), healthy controls (*n* = 184))	16S rRNA sequencing	Five bacterial genera were found in greater abundance (*Porphyromonas*, *Prevotella*, *Corynebacterium_1*, *Bifidobacterium*, and *Lactobacillus*), while ten genera were present in reduced abundance (*Faecalibacterium*, *Agathobacter*, *Blautia*, *Roseburia*, *Fusicatenibacter*, *Lachnospira*, *Butyricicoccus*, *Lachnospiraceae*_ND3007_group, *Lachnospiraceae*_UCG-004, and *Oscillospira*) Classification of bacterial genera into three clusters: (1) opportunistic pathogens (increased in PD), (2) SCFA-producing bacteria (reduced in PD), and (3) Bifidobacterium and Lactobacillus (increased in PD)

This table focuses on the studies on changes in the gut microbiome in the manifest stage of PD. The two studies by Wallen et al. from the years 2020 and 2021 both include two data sets which are the same in both years

PD Parkinson’s disease; SCFA short chain fatty acid; SNCA alpha-synuclein gene; USA United States of America

A study from the United States (*n* = 855) by Wallen et al. aimed to demonstrate the association between PD and gut microbiota composition. The research team conducted a study using two data sets. In both, the microbiome composition of PD patients differed significantly from that of the control group. Five bacterial genera were more abundant in the PD group than in controls (*Porphyromonas*, *Prevotella*, *Corynebacterium_1*, *Bifidobacterium*, and *Lactobacillus*), while ten genera were less abundant in PD patients (*Faecalibacterium*, *Agathobacter*, *Blautia*, *Roseburia*, *Fusicatenibacter*, *Lachnospira*, *Butyricicoccus*, *Lachnospiraceae_ND3007_group*, *Lachnospiraceae_UCG-004*, and *Oscillospira*). These 15 genera were grouped into three clusters: (1) opportunistic pathogens (increased in PD); (2) SCFA-producing bacteria (reduced in PD); and (3) *Bifidobacterium* and *Lactobacillus* (also increased in PD). The researchers noted that the PD-associated microbiome contained opportunistic pathogens (*Porphyromonas* and *Prevotella*), which are normally commensal but may promote infections under certain conditions [17].

Using the same data sets, a subsequent study (*n* = 802) published a year later investigated the interaction between genetic variants in the SNCA region and gut dysbiosis in PD patients.

SNCA is the gene that encodes alpha-synuclein and represents a known risk factor for PD development. Whether the SNCA gene itself leads to microbiome alterations and thereby promotes PD, or whether it causes PD directly and microbiome changes occur secondarily, or whether entirely different factors are involved, remains unclear [18].

In another U.S.-based study (*n* = 724), sequencing of genetic material from a large group of PD patients and healthy controls revealed that over 30% of the tested microbiome parameters differed between the groups, indicating gut dysbiosis. Thirteen of the 15 genera identified by Wallen et al. [17] were statistically confirmed as associated with PD (all except *Oscillospira* and *Prevotella* subgroup). The study further described the PD metagenome as indicative of a disease-promoting microbial profile, enriched with opportunistic pathogens and immunogenic components, dysregulated neuroactive signaling, an abundance of amyloidogenic molecules capable of inducing alpha-synuclein pathology, and overproduction of microbial toxins, accompanied by reduced resilience, characterized by a lower presence of anti-inflammatory and neuroprotective molecules [19].

Another case–control study (*n* = 170) focused on differences in the gut microbiome between PD patients and healthy individuals in rural California. Results showed lower microbial diversity (*p* = 0.04) and altered microbiota composition (*p* = 0.002) in the PD group. PD patients showed increased abundance of three bacterial phyla (*Proteobacteria*, *Verrucomicrobiota*, and *Actinobacteria*) and five bacterial genera (*Akkermansia*, *Enterococcus*, *Hungatella*, and two *Ruminococcaceae* *genera*). In addition, 35 MetaCyc pathways were differentially expressed in PD patients, including pathways related to biosynthesis, degradation/utilization/assimilation, metabolite formation, energy production, and glycan metabolism. The postural instability and gait disorder (PIGD) PD subtype was associated with three bacterial strains and the NAD biosynthesis pathway. PD duration correlated with *Synergistota*, six bacterial genera, and degradation pathways of aromatic compounds. Two bacterial genera were also associated with motor performance [20].

Mehanna et al. conducted a study (*n* = 65) in Egypt examining the gut microbiome of PD patients and healthy controls. PD patients exhibited a significant increase in *Bacteroides* and a significant decrease in *Firmicutes*, the *Firmicutes*/*Bacteroidetes* ratio, and *Bifidobacteria*.

Although *Prevotella* was also reduced in PD patients, this difference was not statistically significant. When comparing clinical PD phenotypes to the control group, the mixed phenotype showed significantly higher *Bacteroides*, while the tremor-dominant phenotype had lower *Firmicutes* and a reduced *Firmicutes*/*Bacteroidetes* ratio. Both tremor-dominant and PIGD phenotypes showed reduced *Bifidobacteria*. A comparison between tremor-dominant and non-tremor phenotypes revealed a significant decrease in Lactobacilli in the non-tremor phenotype [21].

### Alterations of the gut microbiome in correlation with clinical symptoms

In the following section, studies focus on how gut microbiome alterations correlate with clinical symptoms of PD, such as tremor-dominant and non-tremor-dominant subtypes of PD. In addition, the gut microbiome of brain-first and body-first PD is analyzed*.*

A Chinese research team led by Zhang et al. conducted a study (*n* = 200) comparing stool samples from PD patients with those of their healthy spouses (HS) and healthy controls (HP). The microbial composition of the PD, HS, and HP groups differed significantly in both alpha and beta diversity. Microbial composition also varied according to Hoehn and Yahr stage and disease duration. The abundance of eight inflammation-associated microbial genera (*Parabacteroides*, *Akkermansia*, *Coprococcus*, *Bilophila*, *Collinsella*, *Methanobrevibacter*, *Eggerthella*, and *Adlercreutzia*) showed a continuous upward trend with increasing Hoehn and Yahr stage and disease duration, indicating that these genera may be characteristic of advanced PD [22].

A subsequent Italian study (*n* = 56) focused on gut microbiome differences according to PD phenotypes. Participants were categorized into tremor-dominant (TD) and non-tremor-dominant subtypes, the latter including akinetic-rigid and dyskinetic features.

The aim was to assess the gut microbiota and metabolome composition in relation to clinical manifestation. Results revealed a reduction in the relative abundance of *Lachnospiraceae*, *Blautia*, *Coprococcus*, *Lachnospira*, and an increase in *Entero-bacteriaceae*, *Escherichia*, and *Serratia*, which were associated with non-TD subtypes. In addition, concentrations of key molecules (e.g., nicotinic acid, cadaverine, and glucuronic acid) changed in relation to the severity of the phenotype [23].

A study by Park et al. (*n* = 72) from South Korea investigated gut microbiome differences between the body-first and brain-first subtypes of PD. Significant differences in gut microbial composition were observed between body-first PD patients and those with brain-first PD and healthy controls. The microbiome primarily consisted of five phyla: *Firmicutes*, *Bacteroidetes*, *Actinobacteria*, *Proteobacteria*, and *Verrucomicrobia*. The distribution of these phyla accounted for the main differences in beta diversity. Specifically, the gut microbiome of body-first PD patients showed lower levels of *Firmicutes* and *Bacteroidetes*, and higher prevalence of *Actinobacteria*, *Proteobacteria*, and *Verrucomicrobia*, compared to brain-first PD patients and healthy individuals. The microbiome profile of body-first PD was marked by increased presence of *Escherichia coli* and *Akkermansia muciniphila*, alongside a reduction in short-chain fatty acid-producing commensal bacteria. These alterations were accompanied by a higher prevalence of microbial genes related to curli protein biosynthesis and a lower prevalence of genes involved in putrescine and spermidine biosynthesis [24].

The results presented in this paragraph are shown in Table 3.

**Table 3:** Studies on changes in the gut microbiome in correlation with clinical symptoms

Study	Region	Participants	Methods	Results and discussion
Park et al. [24]	South Korea	*n* = 72 (body-first PD (*n* = 15), brain-first PD (*n* = 9, unspecified PD (*n* = 12), healthy controls (*n* = 36)	Deep shotgun sequencing	Significant alterations in the gut microbiome were observed between patients with body-first PD compared to those with brain-first PD The distribution of five major phyla (*Firmicutes*, *Bacteroidetes*, *Actinobacteria*, *Proteobacteria*, and *Verrucomicrobia*) was identified as the source of differences in beta diversity The gut microbiome in body-first PD exhibited a distinct profile characterized by increased abundance of *Escherichia coli* and *Akkermansia muciniphila*, along with a reduced prevalence of commensal bacteria that produce short-chain fatty acids
Vascellari et al. [23]	Europe	*n* = 56 (tremor-dominant PD (*n* = 19), akinetic-rigid PD (*n* = 23), dyskinetic PD (*n* = 14)	16S rRNA sequencing	Decreased relative abundance of *Lachnospiraceae*, *Blautia*, *Coprococcus*, and *Lachnospira*, along with an increase in *Enterobacteriaceae*, *Escherichia*, and *Serratia* Alterations in the concentrations of key molecules (for example, nicotinic acid, cadaverine, and glucuronic acid) in relation to the severity of the phenotype
Zhang et al. [22]	China	*n* = 200 (PD (*n* = 63), healthy spouses (*n* = 63), healthy controls (*n* = 74))	16S rRNA sequencing	The composition of PD, HS, and HP samples differed in terms of both alpha and beta diversity Eight inflammation-associated microbial genera (*Parabacteroides*, *Akkermansia*, *Coprococcus*, *Bilophila*, *Collinsella*, *Methanobrevibacter*, *Eggerthella*, and *Adlercreutzia*) exhibited a continuous upward trend with increasing Hoehn and Yahr stage and longer disease duration

This table gives an overview on studies that focus on changes in the gut microbiome in correlation with clinical symptoms. The results show that alterations in the gut microbiome are different when considering different expressions of PD

*PD* Parkinson’s disease, *HS* healthy spouses, *HP* healthy controls

#### Factors influencing the progression of Parkinson’s disease

To assess whether alterations in the gut microbiome persist over time, a Finnish study by Aho et al. (*n* = 128) compared PD patients with healthy controls, as well as PD patients with stable disease vs. those with a faster disease progression. Significant differences in microbiota composition were found between patients and controls—but not between timepoints. Bacterial taxa that consistently differed between PD patients and controls at baseline and 2 years later included *Roseburia*, *Prevotella*, and *Bifidobacterium*. Differences in taxa associated with disease progression were inconsistent across methods and timepoints; however, there were indications of altered enterotype distribution and a lower abundance of *Prevotella* in patients with faster disease progression. These results suggest that gut microbiota alterations persist even after 2 years in PD patients compared to healthy individuals [25].

Another study by the same research group (*n* = 111) explored the relationship between gut microbiota, intestinal permeability, short-chain fatty acids (SCFAs), and inflammation in PD. Stool and plasma samples, along with clinical data, were collected from PD patients and healthy controls. SCFA levels in stool, along with markers of inflammation and gut permeability in both stool and plasma, were compared between groups and correlated with microbiota profiles.

In PD, stool calprotectin levels were elevated, while SCFA concentrations were reduced, with sex-dependent effects. Inflammatory markers in plasma and stool did not correlate with each other and showed no strong association with SCFA levels. Age at PD onset was positively correlated with SCFA levels and negatively correlated with stool concentrations of CXCL8 and IL-1β. Stool zonulin (gut permeability marker) correlated positively with stool neutrophil gelatinase-associated lipocalin (NGAL, inflammatory marker) and negatively with both motor and non-motor PD symptoms. Microbiota diversity and composition were linked to stool concentrations of SCFAs, inflammatory markers, and zonulin. Some associations differed between PD patients and controls and varied by sex. In PD, inflammatory gut responses and reduced fecal SCFA levels were observed. These were associated with gut microbiota and disease onset but were not reflected in plasma inflammation profiles [26].

A study from Taiwan (*n* = 181) by Chen et al. examined whether and how stool and plasma levels of SCFAs are related to gut microbiota and clinical severity of PD. Compared to healthy controls, PD patients had lower fecal but higher plasma concentrations of acetate, propionate, and butyrate. After adjusting for age, sex, disease duration, and dopaminergic medication dose, motor scores on the MDS-UPDRS part III correlated with lower fecal concentrations of acetate (*p* = 0.012), propionate (*p* = 0.036), and butyrate (*p* = 0.004), and with higher plasma concentrations of propionate (*p* = 0.042). Furthermore, after adjustment cognitive scores (MMSE) showed a negative correlation with plasma butyrate (*p* = 0.027) and valerate (*p* = 0.033). SCFA-producing gut bacteria correlated positively with fecal SCFA levels in healthy controls but showed no such correlation in PD patients. In the PD group, the abundance of pro-inflammatory microbes such as *Clostridiales bacterium* NK3B98 and *Ruminococcus* sp. AM07-15 were significantly associated with reduced fecal SCFAs and increased plasma SCFA concentrations, particularly propionate. In summary, PD patients exhibited reduced SCFAs levels in stool and elevated levels in plasma, both of which correlated with specific microbiota changes and disease severity [27].

These findings are summarized in Table 4.

**Table 4:** Studies on microbiome factors influencing the progression of Parkinson’s disease

Study	Region	Participants	Methods	Results and discussion
Chen et al. [27]	Taiwan	*n* = 181 (PD (*n* = 96), healthy controls (*n* = 85))	Deep shotgun sequencing	Patients with PD exhibited lower fecal but higher plasma concentrations of acetate, propionate and butyrate Motor scores from MDS-UPDRS Part III significantly correlated with reduced fecal concentrations of acetate, propionate and butyrate, as well as increased plasma concentrations of propionate in PD patients
Aho et al. [26]	Europe	*n* = 111 (PD (*n* = 55), healthy controls (*n* = 56))	16S rRNA sequencing	Fecal calprotectin levels were elevated in PD patients, while the concentration of SCFA was reduced in a sex-dependent manner Microbiota diversity and composition were associated with fecal concentrations of SCFA, inflammatory markers and zonulin
Aho et al. [25]	Europe	*n* = 128 (PD (*n* = 64), healthy controls (*n* = 64))	16S rRNA sequencing	Significant differences were observed in the microbiota composition between patients and healthy controls, but not between timepoints (notably involving *Roseburia*, *Prevotella*, and *Bifidobacterium*) Evidence suggested a distinct distribution of enterotypes and a lower abundance of *Prevotella* in PD patients with faster disease progression

This table summarizes the studies that focus on factors influencing the progression of PD

*MDS-UPDRS* Movement Disorder Society Unified Parkinson’s Disease Rating Scale, *PD* Parkinson’s disease, *SCFA* short chain fatty acid

## Discussion

**Fig. 2:**
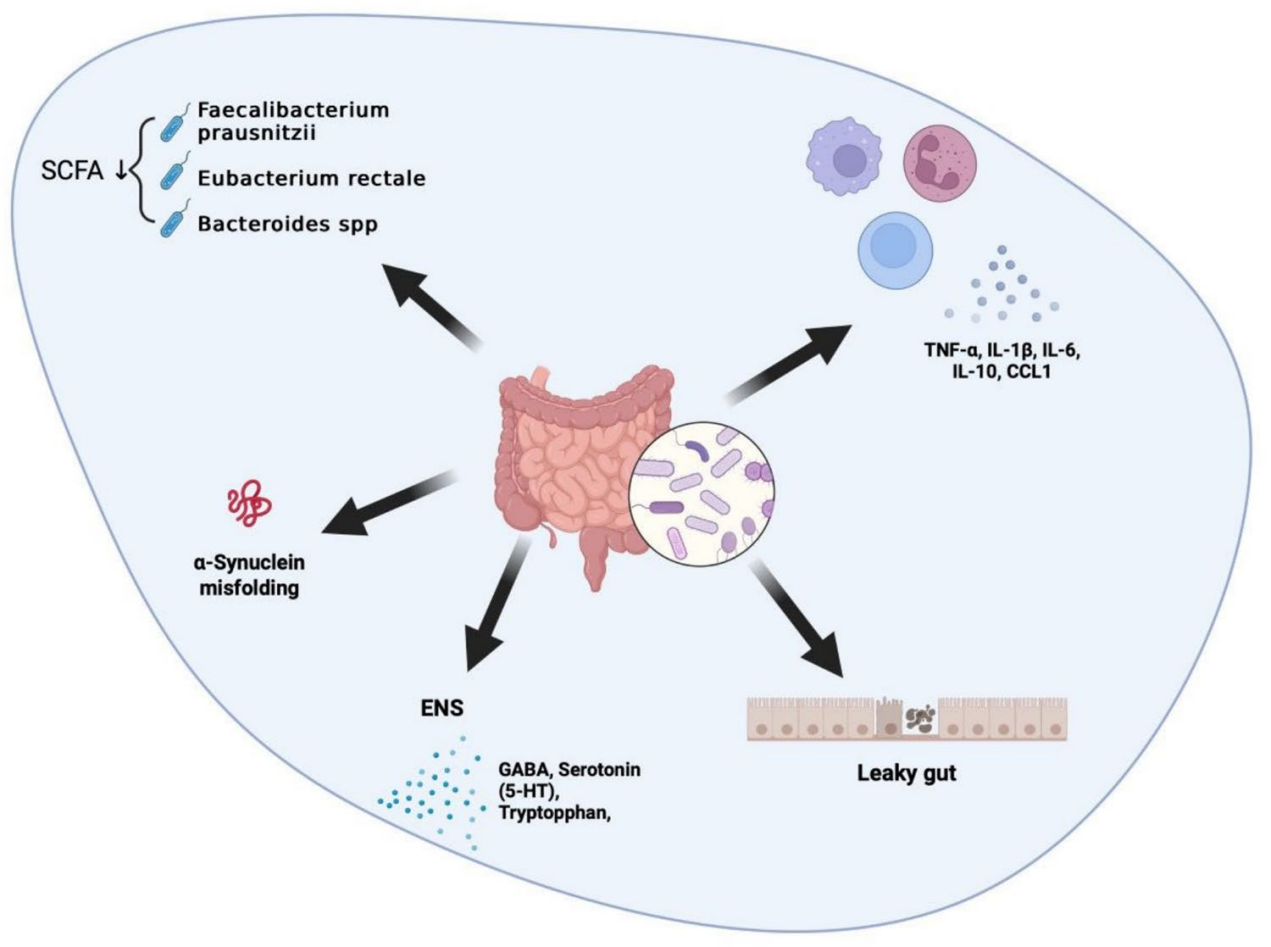
Possible influences of gut dysbiosis on Parkinson’s syndrome. Schematic overview illustrating potential mechanisms by which intestinal dysbiosis may contribute to the pathogenesis of PD. Dysbiosis can lead to a reduction in short-chain fatty acids (SCFAs), which are essential for maintaining intestinal barrier integrity and regulating immune responses. The resulting “leaky gut” allows bacterial products and inflammatory mediators to enter systemic circulation, promoting peripheral and central immune activation. These processes may ultimately affect the enteric nervous system (ENS), facilitating α-synuclein misfolding and propagation along the gut–brain axis, thereby influencing the progression of PD pathology. *CCL* chemokine ligand, *ENS* enteric nervous system, *IL* interleukine, *PD* Parkinson’s disease, *SCFA* short chain fatty acid, *TNF* tumor necrosis factor

The results of this review demonstrate alterations in gut microbiome both in prodromal and clinically manifest stages of PD. Several studies showed correlations between microbial changes and clinical symptoms of the disease. Notably, alterations in the gut microbiota of individuals with prodromal PD appear to follow a continuum between healthy controls, patients with prodromal and manifest PD. These findings collectively suggest a role of gut microbial composition in PD progression. Figure 2 visualizes the most important findings of the studies.

Beyond mere compositional alterations, mounting evidence suggests that inflammatory and metabolic dysregulations within the gut microbiota play a pivotal role in the neuroimmunological mechanisms underlying PD. Several studies have demonstrated a depletion of short-chain fatty acid (SCFA)-producing bacteria, particularly those generating anti-inflammatory metabolites, such as butyrate, acetate, and propionate, in PD patients [17]. From a neuroimmunological perspective, this depletion is highly relevant, as SCFAs exert key modulatory effects on both peripheral and central immune responses. They influence the differentiation and function of regulatory T cells (Tregs), suppress pro-inflammatory cytokine production (e.g., IL-6, TNF, and IL-1β), and enhance the integrity of the intestinal epithelial barrier, thus limiting systemic exposure to bacterial antigens and endotoxins.

The study by Aho et al. further underscores the gut–brain–immune axis as a dynamic bidirectional interface, showing how microbially derived metabolites such as SCFAs regulate not only local gut immunity and permeability but also neuroinflammatory processes within the central nervous system. SCFAs can cross the blood–brain barrier, where they modulate microglial activation states, influence histone acetylation, and thereby impact neuronal function and neuroinflammation.

Concomitantly, PD patients display a systemic dysregulation of inflammatory markers, including elevated IL-6, TNF, IL-1β, CRP, and CCL5, alongside altered levels of anti-inflammatory mediators, such as IL-10 and IL-2, suggesting a shift toward a pro-inflammatory immune phenotype. The reduced abundance of the *Prevotella* enterotype, commonly linked to higher butyrate production and mucin synthesis, supports the hypothesis that microbiota-driven immune imbalances foster chronic inflammation and increased gut permeability, facilitating peripheral immune activation and possibly α-synuclein misfolding in enteric neurons [26].

Moreover, specific microbial community structures associated with non-tremor-dominant PD subtypes may contribute to a sustained pro-inflammatory intestinal milieu. This, in turn, could exacerbate gastrointestinal dysfunction, promote enteric glial activation, and potentiate prion-like propagation of misfolded α-synuclein along the vagal pathway, ultimately linking peripheral immune dysregulation to central neurodegeneration [23].

Alpha-synuclein is widely recognized as a key contributor in PD pathogenesis. The SNCA gene, which encodes alpha-synuclein, has been shown to increase alpha-synuclein expression after infections unrelated to PD, such as gastrointestinal infections [28]. Based on these findings, researchers hypothesized that if opportunistic pathogens are involved in disease pathogenesis, there might be an interaction between genetic variants in the SNCA region and dysbiosis of the gut in PD. The trigger that induces alpha-synuclein pathology in the gut is unknown. A link between SNCA expression and the presence of opportunistic pathogens needs to be further investigated [18].

The potential bidirectional interaction between PD medications and the gut microbiome was addressed in several studies [14, 17, 25]. Most participants in these studies were receiving dopaminergic treatment. Several studies examined the impact of the commonly used PD drug Levodopa on the gut microbiome and vice versa. Genes from gut microbes such as *Enterococcus faecium* and *Enterococcus faecalis* encode tyrosine decarboxylase, which metabolizes Levodopa to dopamine in the gut before it enters the brain. A higher abundance of these bacteria was associated with reduced Levodopa efficacy. Both *E. faecium* and *E. faecalis* were found in elevated amounts in stool samples from PD patients [19]. However, other studies found no association between alpha or beta diversity and Levodopa dose, nor between Levodopa dose and specific microbial taxa [20]. Similarly, another study found no significant differences in microbiota composition between PD patients on different medications or on high (> 400 mg/day) vs. low (≤ 400 mg/day) Levodopa doses [22]. In general, it remains difficult to determine whether a given drug alters the microbiome or whether the microbiome modulates drug effects. Nonetheless, the gut microbiome plays a role in drug metabolism, which requires further investigation [29–31].

The potential for pathogens to spread from the gut to the brain via the vagus nerve has gained increasing attention. Epidemiological studies and animal models offer preliminary insights in this regard. To investigate whether a pathogen could potentially enter the central nervous system via the vagus nerve, Svensson et al. studied patients who had undergone vagotomy. They found that patients with a truncal vagotomy had a lower risk of developing PD compared to those with a super selective vagotomy and the general population. Patients who had undergone super selective vagotomy had a similar PD risk as the general population. Overall, truncal vagotomy was associated with a lower risk of PD, suggesting that the transmission of pathogenic agents via the vagus nerve might be involved in PD pathogenesis [32]. A cohort study investigating the incidence of PD following vagotomy in the Swedish population similarly found no general association between vagotomy and PD risk but did report a reduced risk in patients with truncal vagotomy [33]. An animal model provided further support for Braak’s hypothesis, which posits that pathological alpha-synuclein can spread from the gastrointestinal tract to the brain via the vagus nerve [10]. In this study, mice were injected with pathological alpha-synuclein into the duodenal and pyloric muscle layers, and the spread of the protein into the brain was observed. Over time, dopaminergic neuronal loss and both motor and non-motor symptoms developed. Truncal vagotomy and alpha-synuclein deficiency prevented the spread of the pathology from the gut to the brain and the resulting neurodegeneration and behavioral deficits. These results suggest that the propagation of alpha-synuclein from the gut to the central nervous system may contribute significantly to PD pathogenesis and support the Braak hypothesis [34, 35].

Several emerging therapeutic strategies aim to modulate the gut microbiota in PD. These include increasing dietary fiber intake to promote microbial diversity, targeted probiotic supplementation with selected bacterial strains and fecal microbiota transplantation to treat dysbiosis in PD patients [36]. As already pointed out a pro-inflammatory microbiome plays an important role in PD. The study by Hall et al. investigated the effects of a prebiotic high-fiber diet on the gut microbiota. These findings indicate that increased fiber intake promotes the production of beneficial metabolites, such as SCFAs. The intervention was well-tolerated, considered safe for PD patients and was further associated with a reduction of gastrointestinal symptoms. In addition, anti-inflammatory shifts in the microbiota were observed as well as reduced levels of calprotectin and zonulin, which are markers of intestinal inflammation and barrier dysfunction [37]. In the following study patients with PD received a symbiotic treatment (*Enterolactis Duo*, containing the probiotic strain *Lacticaseibacillus paracasei* DG and the prebiotic fiber inulin) for 12 weeks and were assessed before and after to evaluate benefits of this therapy. After 12 week constipation and other non-motor symptoms improved in PD patients. In addition, changes in the gut microbiome were identified as well as an increase of SCFAs. This study reveals that probiotic supplementation may play an important role in PD treatment [38]. Another potential approach to modulating the gut microbiota in patients with PD may be fecal microbiota transplantation (FMT). A randomized, placebo-controlled trial investigated the effects of FMT in PD patients compared to a placebo group. Patients receiving the intervention showed significant improvements in PD-related motor symptoms as well as autonomic symptoms, reduction of gastrointestinal complaints, and an increased microbial richness [39]. Although these therapies show first promising results and seem to help PD patients, larger studies are needed to confirm efficacy of these therapeutic approaches.

In addition to the aforementioned intervention strategies, diet also plays a crucial role in influencing the progression of PD. The study by Hegelmaier et al. demonstrated that PD patients who followed an ovo-lacto vegetarian diet for a period of 14 days and received SCFAs exhibited a modified gut microbiome compared to baseline. A subset of these patients also received a daily fecal enema. UPDRS Part III scores improved significantly, and the levodopa-equivalent daily doses was reduced over the course of 1 year following the dietary intervention and regular fecal enemas. Furthermore, a significant association between gut microbiome diversity, UPDRS Part III scores, and the abundance of *Ruminococcaceae* was observed. The abundance of *Clostridiaceae* was significantly reduced after the enemas. These findings highlight that dietary modifications and bowel cleansing may represent an additional non-pharmacological therapeutic option for patients with PD optimizing enteral resorption of PD drugs [40].

Several limitations of the reviewed studies must be acknowledged. Most notably, all included studies assessed gut microbiota via stool samples, typically using 16S rRNA sequencing. While this method is widely used, it does not capture viral or fungal components, which could also be relevant. Moreover, differences in laboratory protocols, sample handling (e.g., collection, storage, and transport), and statistical methods can significantly influence results, making direct comparisons across studies difficult [17]. Standardized international protocols are, therefore, essential. Further limitations include variation in study objectives, small sample sizes, and short follow-up durations. Future studies should aim to address these issues using larger cohorts and long-term follow-ups to further explore the gut microbiome’s role in PD [25].

Moreover, diet is a major determinant of gut microbiome composition and, therefore, represents an important potential confounder in microbiome research. In the studies included in our review, dietary information was not assessed uniformly, which limits the ability to account for nutrition-related variability across cohorts. The questionnaires employed in some studies included the Gut Microbiome Questionnaire (GMQ), the Family History Questionnaire (EFQ), the Diet History Questionnaire II (DHQ II), and various Food Frequency Questionnaires (FFQ). Additional lifestyle-related confounders, such as BMI, alcohol consumption, smoking status, medical history, and medication, were also considered in several studies, although inconsistently. Future research would benefit from implementing standardized dietary assessment procedures to more reliably account for the impact of nutrition on gut microbiome profiles in PD [41]. These findings are summarized in Table 5.

**Table 5:** Potential confounders related to diet and lifestyle in study population

Study	Diet information
Wallen et al. [17]	Geography, BMI, alcohol use, fruits or vegetables daily, GI health, medications (GMQ, EFQ)
Wallen et al. [18]	Geography, BMI, alcohol use, fruits or vegetables daily, GI health, medications (GMQ, EFQ)
Wallen et al. [19]	Geography, BMI, diet (fruits or vegetable, meat, nuts, yogurt, and grains), alcohol use, cigarette and caffeine use, GI health, medications (GMQ, EFQ)
Huang et al. [14]	Exclusion criteria: antibiotics usage within 1 month, pre-existing gastrointestinal diseases that confound gut microbiota (e.g., inflammatory bowel diseases and liver cirrhosis) Geography, lifestyle (e.g., smoking, coffee drinking, and exercise), GI health
Zhang et al. [20]	Standardized interviews (demographic information, medical histories including medications) (DHQ II)
Aho et al. [26]	BMI, medical history, medication, diet
Aho et al. [25]	BMI, medical history, medication, diet
Vascellari et al. [23]	BMI, coffee consumption, smoking status, medication All patients followed a well-balanced Mediterranean diet without nutritional variations (according to Mediterranean diet questionnaire) Exclusion criteria: primary gastrointestinal disease, use of probiotics or antibiotics in the 3 months before enrolment
Chen et al. [27]	BMI, medication, medical history, dietary information (Food Frequency Questionnaire (FFQ))
Palacios et al. [13]	BMI, medical history, lifestyle (smoking status, pack years smoking, alcohol and caffeine intake), dietary information (FFQ), the Mediterranean Diet Score based on the FFQ was used as an aggregate measure of diet quality
Park et al. [24]	Medical history, medication
Heintz-Buschart et al. [15]	BMI, medical history, medication, smoking status
Mehanna et al. [21]	Exclusion criteria: conditions that independently affect the gut microbiome, such as severe renal or liver impairment, inflammatory bowel disease and recent antibiotic use
Zhang et al. [22]	Exclusion criteria: inflammatory bowel disease, diabetes, gastrointestinal disease, surgical history, infectious diseases, antibiotics/probiotics used for nearly 3 months Medical history, medication (interviews)
Zhang et al. [16]	BMI, medical history, medication, smoking status, alcohol use Exclusion criteria: vegetarian or malnutrition, history of chronic gastrointestinal disorder or surgery, regular consummation of yogurt and use of 19 medications that were reported to have significant association with microbiota

This table summarizes information taken in clinical assessment as well as dietary information of the patients that potentially have impact on the gut microbiome

*BMI* body mass index, *DHQ II* Diet History Questionnaire II, *EFQ* Family History Questionnaire, *GI* gastrointestinal, *GMQ* Gut Microbiome Questionnaire

Especially regarding diagnostic and therapeutic applications, the microbiome represents a promising area of research for this still incurable disease with largely unknown origins.

Furthermore, this review has some limitations that must be considered. Only studies with at least 30 patients per group were included. Future reviews should include broader sample sizes and aim for comprehensive meta-analyses to strengthen generalizability.

## Conclusion

This systematic literature review examined how the gut microbiome changes in PD, over the disease course and how it correlates with clinical symptoms. Based on the data from the included studies, it can be concluded that the gut microbiome of PD patients differs significantly from that of healthy controls. Whether these alterations are a causal factor in the development of this neurodegenerative disease or a consequence of the disease itself remains unclear and requires further investigation. In general, advances in microbiome analysis techniques will likely allow a more precise investigation in the future. Ongoing research may identify microbial biomarkers that could provide valuable insights into the diagnosis, progression, and severity of PD. A major focus of future studies should also be the development of new therapeutic strategies for PD patients by targeting the gut microbiome.

## Supplementary Information

Below is the link to the electronic supplementary material.

Supplementary file1 (DOCX 119 KB)

Supplementary file2 (PDF 144 KB)

## Data Availability

This is a systematic review article, so we do not have original data to share. The main findings of this study were displayed in the tables.
