# Supplementary material for: The role of the gut microbiome in the progression of Parkinson’s disease: a systematic review of patient cohorts
Source: J Neurol. 2025 Dec 6;273(1):8. doi: 10.1007/s00415-025-13545-8 (PMC12681464; doi:10.1007/s00415-025-13545-8)
Supplement: Supplementary file 1 — Supplementary file1 (DOCX 119 KB) [file 415_2025_13545_MOESM1_ESM.docx]

**Supplementary Table 1: QUADAS-2 score**

| Study | Risk of Bias |  |  |  | Concerns regarding applicability |  |  |
| --- | --- | --- | --- | --- | --- | --- | --- |
|  | Patients Selection | Index Test | Reference Standard | Flow and Timing | Patients Selection | Index Test | Reference Standard |
| Wallen et al. (2020) | ? | ? | ? | + | ? | + | + |
| Wallen et al. (2021) | ? | ? | ? | + | ? | + | + |
| Huang et al. (2023) | - | ? | + | + | + | + | + |
| Wallen et al. (2022) | ? | - | + | ? | - | + | + |
| Zhang, K et al. (2022) | ? | - | + | + | ? | + | + |
| Aho, V et al. (2021) | - | - | + | + | ? | + | + |
| Chen et al. (2022) | ? | - | + | + | + | + | + |
| Palacios et al. (2023) | - | - | ? | + | + | + | - |
| Park et al. (2024) | - | - | + | + | + | + | - |
| Heintz-Buschart et al. (2018) | - | - | + | + | + | + | + |
| Mehanna et al. (2023) | ? | - | + | + | + | + | + |
| Aho, V et al. (2019) | - | - | + | ? | + | + | + |
| Zhang, F et al. (2020) | ? | - | + | + | + | + | + |
| Vasceralli et al. (2021) | + | - | + | + | + | + | + |
| Zhang, P et al. (2024) | ? | ? | + | + | + | + | + |

This table shows the risk of bias of the used studies using the QUADAS-2 score.
